# High-Performance Thermal Interface Materials with Magnetic Aligned Carbon Fibers

**DOI:** 10.3390/ma15030735

**Published:** 2022-01-19

**Authors:** Qi Wu, Jianyin Miao, Wenjun Li, Qi Yang, Yanpei Huang, Zhendong Fu, Le Yang

**Affiliations:** 1 Beijing Key Laboratory on Space Thermal Control Technology, Beijing Institute of Spacecraft System Engineering, Beijing 100094, China; wuqicn@outlook.com (Q.W.); hitmmc@hotmail.com (W.L.); yangqi_cast@163.com (Q.Y.); hypbuaa@buaa.edu.cn (Y.H.); fuzd1988@163.com (Z.F.); leletianxu@hotmail.com (L.Y.); 2 School of Aeronautic Science and Engineering, Beihang University, Beijing 100191, China

**Keywords:** thermal interface material, milled carbon fibers, magnetic alignment, thermal conductivity, hardness

## Abstract

Thermal interface materials with high thermal conductivity and low hardness are crucial to the heat dissipation of high-power electronics. In this study, a high magnetic field was used to align the milled carbon fibers (CFs, 150 μm) in silicone rubber matrix to fabricate thermal interface materials with an ordered and discontinuous structure. The relationship among the magnetic field density, the alignment degree of CFs, and the properties of the resulting composites was explored by experimental study and theoretical analysis. The results showed higher alignment degree and enhanced thermal conductivity of composites under increased magnetic flux density within a certain curing time. When the magnetic flux density increased to 9 T, the CFs showed perfect alignment and the composite showed a high thermal conductivity of 11.76 W/(m·K) with only 20 vol% CF loading, owing to the ordered structure. Meanwhile, due to the low filler loading and discontinuous structure, a low hardness of 60~70 (shore 00) was also realized. Their thermal management performance was further confirmed in a test system, revealing promising applications for magnetic aligned CF–rubber composites in thermal interface materials.

## 1. Introduction

As a common type of thermal management material, thermal interface material (TIM) has been extensively applied in electronic packaging to fill the gap between the contact interface of heat sources and heat sinks, which constitutes a dominant factor in achieving efficient thermal transfer. Besides high intrinsic thermal conductivity, low hardness is also crucial to achieving high performance in TIMs, which means that the TIMs should be able to deform to conform to the topography of the mating surface so as to improve interfacial heat transfer, and is also especially vital when working with irregular surfaces [1,2,3]. However, it is difficult to achieve these properties simultaneously. There is no denying that the thermal conductivity of composites can be improved by increasing filler content [4], but this comes at the expense of increased hardness [5]. Therefore, reasonable microstructure with low filler loading is necessary to achieve high-performance TIMs [6].

It is generally accepted that filler alignment is an effective approach to remarkably improve the thermal conductivity of composites in a specific direction [4,6], which is useful and well suited to the requirements of TIMs. The control over filler alignment has been used to make the most of the advantages of the anisotropic properties of low-dimensional materials. In terms of thermal properties, the axial thermal conductivity of one-dimensional fillers and the in-plane thermal conductivity of two-dimensional fillers are usually much higher than those in the perpendicular direction. Based on this, some research efforts have been directed towards the alignment of alumina platelet [7,8], boron nitride [9,10,11], carbon nanotube [12,13,14,15], carbon fiber (CF) [5,16,17], graphene [18,19,20,21] and some other fillers. Among these fillers, CF has greater potential due to its higher axial thermal conductivity (up to 1000 W/(m·K)) compared to ceramic fillers (alumina platelet, boron nitride), and its more appropriate size than nanofillers (carbon nanotube, graphene), which have more filler/polymer interface, resulting in phonon scattering. In some of the previous studies, the CFs were long enough to penetrate through the material, which significantly improved the thermal performance of TIMs [5]. However, when these penetrating CFs are compressed, they have no room to move downward, making it difficult for the TIM to deform in the thickness direction. Conversely, the discontinuous structure composed of shorter fibers allows TIM to take advantage of the high elasticity of the matrix to produce greater deformation and lower hardness. Therefore, milled CF is a more suitable filler for TIM.

Several approaches have been proposed to obtain aligned fillers, and alignment under external forces is quite common, such as shearing and gravitational force, which can be obtained by extrusion molding [16,22,23], injection molding [24,25,26,27], tape casting [28,29,30], electrospinning [9,31,32], sedimentation [33,34], etc. However, in the process of external force alignment, the force on the fibers at different locations is different, which may lead to a three-layer or even five-layer structure inside the composite, with different degrees of orientation within different layers [35,36]. Thus, external field alignment is more preferable in this regard, because the external field applies the same force to the fibers at different locations, which helps to prepare composites with high uniformity and high orientation degree. As a typical external field, magnetic field is a promising way to control the alignment of fillers in a non-contact and non-destructive process, and is attracting increasing attention. Most thermal conductivity fillers have weak magnetism. Based on this, the methods of aligning these fillers by magnetic field can be divided into two types. The first is coating the fillers with strongly magnetic nanoparticles to achieve an ultrahigh magnetic response to a low external magnetic field [7,37,38,39,40]. Using a superconducting magnet is another to realize the alignment [41]. The high magnetic flux density generated by the superconducting magnet can control feebly magnetic fillers without the help of other mediums. Although it is an appealing method, there are relatively few studies devoted to aligning high-thermal-conductivity CF directly using a high magnetic field, and experimental studies of the impacts of magnetic flux density on microstructure and properties of CF reinforced composites are even fewer. In most experimental studies of magnetic alignment, magnetic flux density was a fixed value. Usually, only the oriented structure and disordered structure are compared, and the state between the two is rarely investigated.

In this paper, milled mesophase pitch-based CF was selected as the filler of polymer composites due to its high axial thermal conductivity and appropriate size. An ordered and discontinuous structure was achieved by the alignment of milled CFs with a high magnetic field. The through-plane thermal conductivity of the composites was dramatically enhanced because of the ordered microstructure created by superconducting magnet. Through changing the magnetic flux density, the influence of magnetic flux density on the microstructure and properties of composites was investigated. The reasons for the differences between the experimental results and theoretical calculations regarding magnetic alignment were also analyzed. Owing to the low filling fraction and discontinuous structure, the composites also displayed satisfactory performance in hardness. In addition, the composites with magnetic alignment showed outstanding thermal management performance when they were used as TIMs.

## 2. Materials and Methods

### 2.1. Materials

The milled CFs used in this paper are mesophase pitch-based CFs with an average length of 150 μm and axial thermal conductivity of 900 W/(m·K). The silicone rubber used in this paper is addition-curing and two-part silicone rubber with low viscosity of 1 Pa·s which is beneficial for the filler alignment and its pot life (up to 100 Pa·s) is about 4 h at room temperature.

### 2.2. Preparations

**Figure 1:**
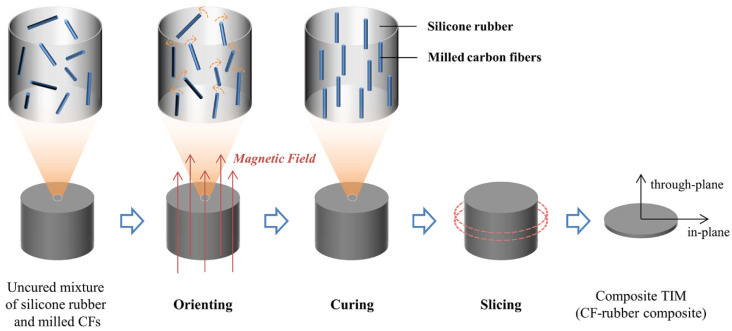
Schematic illustration of the preparation process of CF–rubber composites.

The TIMs in this study were fabricated by magnetic alignment. In a typical process, a certain mass of CFs was added to silicone rubber and then mixed in a planetary centrifugal mixer. To lessen the crosslinks between component A and B of silicone rubber before alignment, the CFs were mixed with component A and B, respectively, and then they were mixed for about 4 min at 1000 rpm to form the final mixture. The bubbles introduced during the mixing process were removed by deaerating for 2 min. After that, 50 g of uncured mixture in mold (50 mm in diameter) was immediately placed in the center of the vertical room temperature bore of a cryogen-free 9 T superconducting magnet (Cryomagnetics Inc., Oak Ridge, TN, USA) until fully cured (at least 4 h), and the magnetic field direction was parallel to the thickness direction of the sample. TIMs (i.e., CF–rubber composites) with required thickness were achieved by ultrasonic cutting along the in-plane direction. It should be mentioned that the samples used for in-plane thermal conductivity measurements were obtained by cutting along the through-plane direction. Reference samples prepared without magnetic alignment were fabricated for comparison. The preparation process is schematically illustrated in Figure 1, and the changes in internal structure of the composite are also shown in this figure. In the uncured mixture, the CFs were randomly distributed in silicone rubber. During the orientation process, the CFs gradually rotated to the direction of the magnetic field and this orientation structure was fixed when the curing was completed.

### 2.3. Characterizations

Scanning electron microscopy (SEM, Quanta FEG 250, FEI, Hillsboro, OR, USA) was used to characterize the micro-morphology of CF–rubber composites at an accelerating voltage of 10 kV. To achieve better imaging quality, samples were sputter coated with a thin layer of gold before visualization. To evaluate the alignment degree of milled CFs in polymer matrix, X-ray diffraction (XRD) analysis of CF–rubber composites was carried out with a Rigaku SmartLab X-ray diffractometer (Rigaku, Tokyo, Japan) using Cu Kα radiation of 1.54 Å at a generator voltage of 40 kV with reflection mode. The thermal conductivity (*λ*, W/(m·K)) was calculated as the multiplication of thermal diffusivity (*α*, mm^2^/s), density (*ρ*, g/cm^3^), and specific heat capacity (*C_p_*, J/(g·K)). The thermal diffusivity (*α*) of the specimens (with a dimension of Φ12.7 × 1 mm) was measured by the laser flash method using an LFA447 Nanoflash (NETZSCH Instruments, Selb, Germany). The density (*ρ*) was measured by Archimedes’ principle, and the specific heat capacity (*C_p_*) was measured using differential scanning calorimetry (DSC 214, NETZSCH Instruments, Selb, Germany). The hardness was measured using a hardness tester (Shore 00, Instron, Norwood, MA, USA). The thermal management performance of composites was characterized by an infrared thermal imager (E8xt, FLIR, Wilsonville, OR, USA). In the test, a ceramic heating plate with 7 W power and a cold plate at 15 °C acted as the heat source and heat sink, respectively. The CF–rubber composite was sandwiched between two aluminum plates that were connected with the heating plate and the cold plate, respectively. The composites were tested as TIMs in this test system.

## 3. Results and Discussion

### 3.1. Alignment of CFs in the CF–Rubber Composites

**Figure 2:**
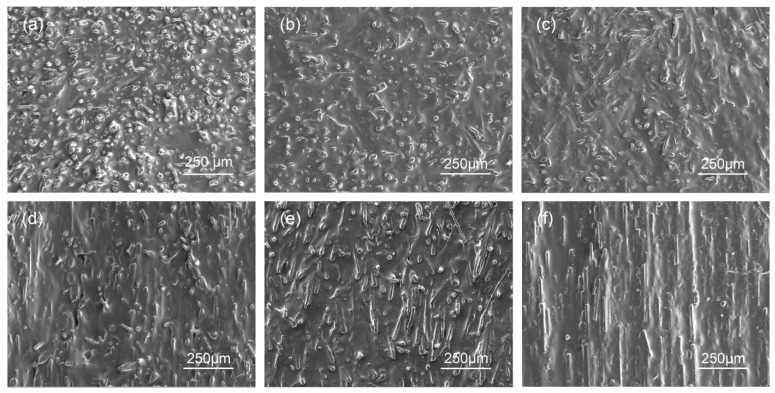
SEM vertical-section images of CF–rubber composites applied with different magnetic flux density. (**a**) 0 T (**b**) 1 T (**c**) 3 T (**d**) 5 T (**e**) 7 T (**f**) 9 T.

The CF–rubber composites were prepared by applying a magnetic field with different magnetic flux densities (1 T, 3 T, 5 T, 7 T, and 9 T). A composite without applying a magnetic field (0 T) was employed as a reference sample. Figure 2 shows the SEM vertical-section images of 20 vol% CF–rubber composites. It was found that CFs tended to align along the in-plane direction when no magnetic field was applied (Figure 2a), which is consistent with the observations in some previous studies [7,41]. The reason for this is that fillers with high aspect ratio tend to align with the substrate under the influence of gravity. The magnetic alignment in the 1 T sample is not obvious (Figure 2b). When the magnetic flux density is increased to 3 T, the CFs start to indicate some preference for alignment along the direction of the magnetic field. With the increase in magnetic flux density, this preference becomes more and more apparent (Figure 2c–f). As shown in Figure 2, the CFs of the sample exposed to 9 T display better alignment than the other samples at lower magnetic flux density.

**Figure 3:**
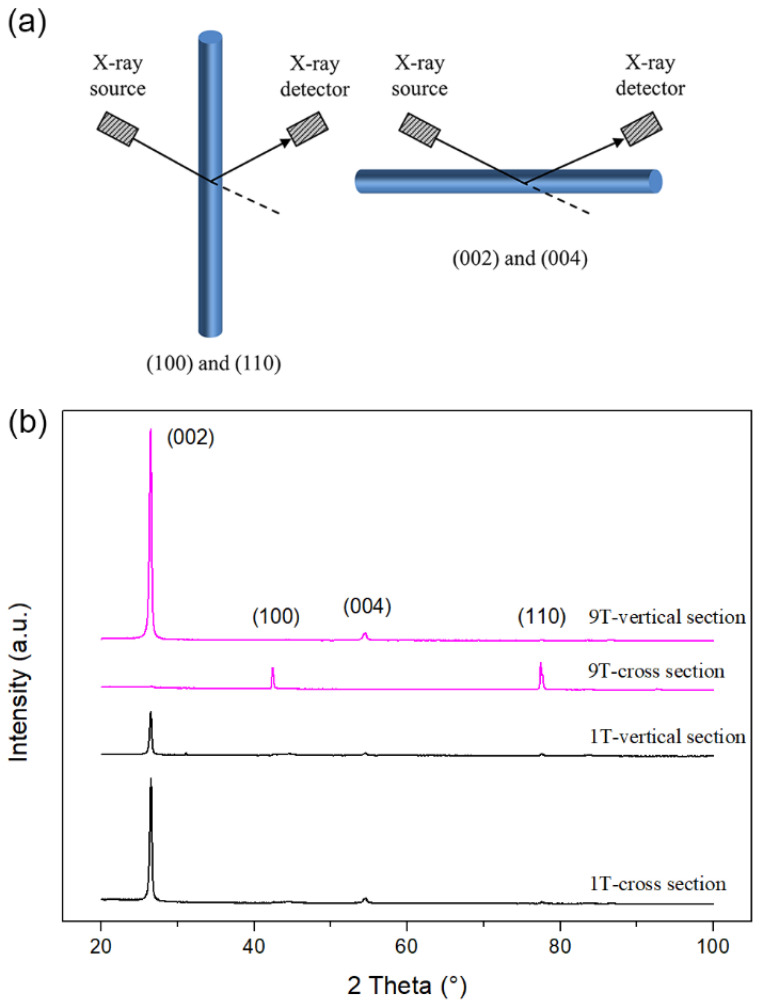
XRD analysis of CF–rubber composites. (**a**) Illustration of alignment effect of CFs on XRD patterns; (**b**) XRD patterns of 1 T and 9 T samples in vertical-section and cross-section.

This trend can also be confirmed by XRD analysis. As schematically illustrated in Figure 3a, the vertically and horizontally aligned CFs are responsible for the (100), (110) peaks and the (002), (004) peaks, respectively. The XRD patterns in Figure 3b show that the peak intensities for the CF–rubber composites at different magnetic flux densities are dramatically different and all have obvious anisotropy. When the magnetic flux density was 1 T, the (002) peak was much stronger than (100) and (110) peaks and the (002) peak in cross-section was stronger than that in vertical-section. When the magnetic flux density was increased to 9 T, the (002) and (004) peaks in vertical-section became stronger; meanwhile, these two peaks almost disappeared in cross-section, and the (100) and (110) peaks were strong, which was the opposite of the 1 T sample. These changes in XRD patterns lead us to conclude that the better filler alignment in the through-plane direction is associated with higher magnetic flux density, which is consistent with the observations from the foregoing SEM images. To further clarify the experimental phenomenon, the alignment degree of CFs (*δ*) was quantified by comparing the relative intensities (*I*) of the (100), (110) peaks in cross-section to the sum of the relative intensities (*I*) of the (100), (110), (002) and (004) peaks in cross-section.

(1) $$\delta =\frac{I_{100}+I_{110}}{I_{100}+I_{110}+I_{002}+I_{004}}\times 100\%$$

The computed results of *δ* for the CF–rubber composites at different magnetic flux densities are summarized in Table 1. It can be seen that the *δ* values of the samples exposed to high magnetic flux density are much higher, which demonstrates that these samples have a greater proportion of through-plane-aligned CFs. The *δ* of 0 T sample and 1 T sample are almost the same, which is consistent with Figure 2, and the slightly higher *δ* of 0 T sample than that of 1 T sample may be due to experimental or test errors.

**Figure 4:**
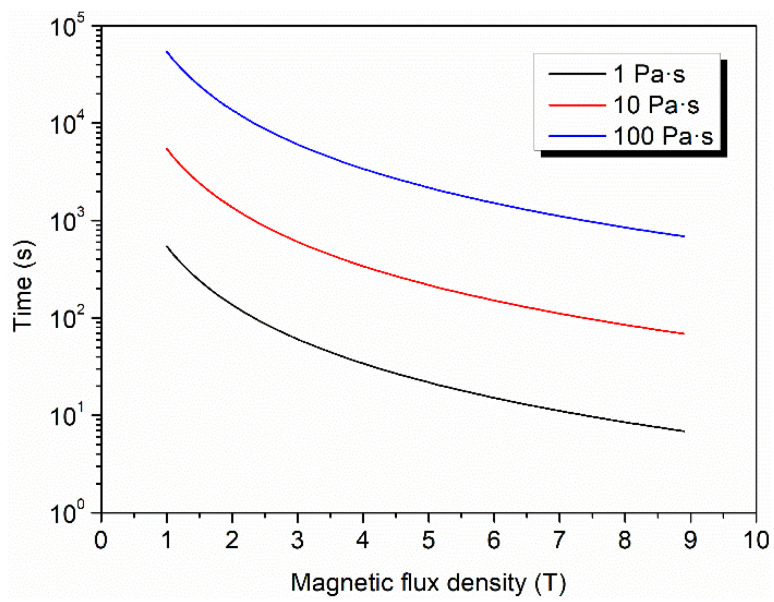
The effect of viscosity on alignment time as a function of magnetic flux density.

To trace the effects of magnetic flux density on the alignment of CFs, a theoretical analysis was carried out. According to the theory of Kimura [42], the alignment of a fiber in a magnetic field is determined by the balance of the magnetic torque and the hydrodynamic torque. The hydrodynamic torque is a function of the rotation speed of the fiber; thus, the alignment time can be calculated based on the parameters of fiber, matrix, and magnetic field. It is noteworthy that according to this theory, a fiber with anisotropic magnetic susceptibility can be aligned even if the magnetic flux density is low, but the alignment time would be very long, so it seems that the fiber cannot be aligned by low magnetic field in practice. The predicted alignment time of CF in matrix with different viscosity is shown in Figure 4 (see details in Supplementary Material). The viscosity of the silicone rubber used in this study is about 1 Pa·s and the predicted alignment time is only 545 s when the magnetic flux density is only 1 T, which is much less than the curing time. Contrary to expectation, the alignment degree of CFs in CF–rubber composites with 1 T magnetic field is low. A possible explanation is that this theory is a study on a single fiber. When a mass of fibers are added to the matrix, the integrated viscosity of the mixture rise drastically. Alternatively, the viscosity of the silicone rubber is gradually increased during the curing. Such factors could account for the difference between the theory and the experiment. As shown in Figure 4, when the viscosity increases to 100 Pa·s, the required alignment time in 1 T magnetic field is over fifty thousand seconds, which is far more than the curing time, and this means that the CFs cannot complete the alignment before the silicone rubber is fully cured. Additionally, at the same viscosity, if the magnetic flux density is increased to 9 T, the required alignment time would decrease dramatically to about 700 s. On the basis of these findings, it can be concluded that magnetic flux density is the key to improving the alignment in practice.

**Figure 5:**
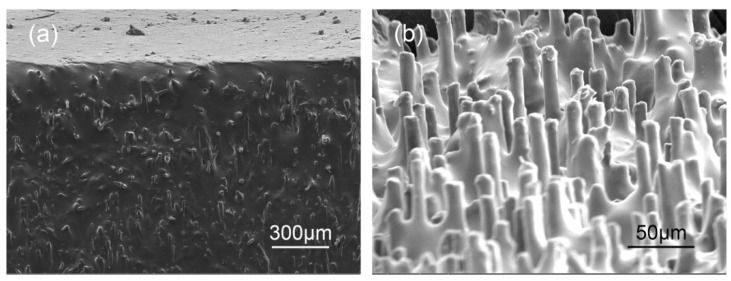
SEM images of the surface of the CF–rubber composites. (**a**) Before slicing (**b**) After slicing.

As can be seen from Figure 5a that, due to the restriction of the wall surface of mold, the surface of the sample before the treatment of slicing is mainly covered with silicone rubber and the CFs near the surface are disordered. We hence obtain specimens with a certain thickness by slicing rather than direct molding. The surface morphology of the 9 T sample after slicing is shown in Figure 5b. It is obvious that the CFs are exposed on the surface in good order, which is beneficial for the thermal conductivity at interface when the composites are used as TIMs.

### 3.2. Thermal and Mechanical Properties of the CF–Rubber Composites

**Figure 6:**
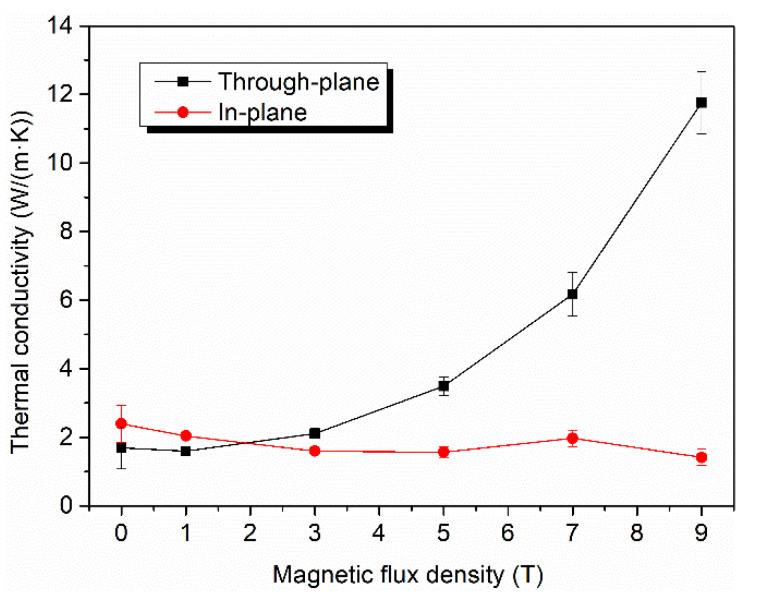
The through-plane and in-plane thermal conductivity of the CF–rubber composites as a function of magnetic flux density.

**Figure 7:**
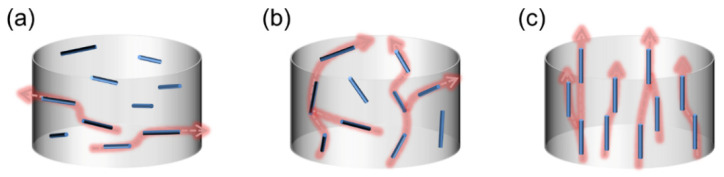
The effect of the alignment degree of CFs on theoretical through-plane thermal conductivity of the CF–rubber composites. (**a**) In-plane alignment; (**b**) random alignment; (**c**) through-plane alignment.

**Figure 8:**
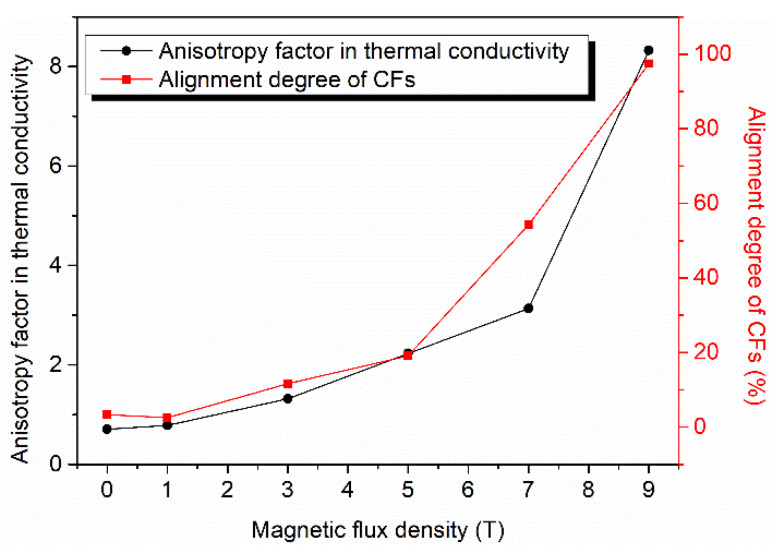
The anisotropy factor in the thermal conductivity of the CF–rubber composites and the corresponding alignment degree of the CFs.

The thermal conductivity of the CFs used in this study has obvious anisotropy. The axial thermal conductivity of these CFs is as much as 900 W/(m·K), but the radial thermal conductivity is only around 5 W/(m·K), with a difference of up to 180 times. Therefore, the alignment of CFs is crucial to the thermal properties of the composites. The thermal conductivity of the CF–rubber composites in both the through-plane and in-plane directions is shown in Figure 6 as a function of magnetic flux density. The thermal conductivity of composite without applying magnetic field (0 T) was also measured for comparison. It is apparent that the composites acquired enhanced thermal conductivity in the through-plane direction with increased magnetic flux density. With the increase in magnetic flux density from 0 T to 9 T, the thermal conductivity in the through-plane direction increased nearly 6-fold, rising from 1.69 W/(m·K) to 11.76 W/(m·K). In contrast, the thermal conductivity in the in-plane direction followed a downward trend. Such tendencies could account for the rotation of CFs in the magnetic field. According to the foregoing SEM images and XRD patterns, when the applied magnetic flux density is low, the axis of CFs with higher thermal conductivity tends to array along the in-plane direction, and thus the in-plane thermal conductivity is higher than the through-plane thermal conductivity, as can be seen from the data points of the 0 T and 1 T samples in Figure 6. When the applied magnetic flux density is higher, the CFs start to overcome the restriction of gravity and align along the direction of the magnetic field, namely in the through-plane direction. Therefore, the through-plane thermal conductivity increases, and the in-plane thermal conductivity decreases. Since the through-plane alignment is in a single direction rather than along the plane, and the in-plane alignment of the 0 T sample is incomplete, the through-plane thermal conductivity of the 9 T sample is much higher than the in-plane thermal conductivity of the 0 T sample, and the improvement of through-plane thermal conductivity with increasing magnetic flux density is much more remarkable than the decline of in-plane thermal conductivity. These results can be attributed to the thermal conductive chains created by CFs. The heat tends to be transferred through the axis of CFs owing to the anisotropic thermal property of CFs and so the axis of CFs is the key constitution of the thermal conductive chains. As illustrated in Figure 7, when the CFs are aligned along the in-plane direction, thermal conductive chains also tend to align along this direction. On the contrary, when the CFs are aligned along the through-plane direction, not only does the length of the thermal conductive chains in the through-plane direction decrease visibly, the thermal conductive chains created by the same number of CFs are also more numerous. Hence, the magnetic alignment of CFs confers a much better thermal transfer performance along the CF alignment direction. To trace the correlation between the anisotropy in the thermal conductivity of the CF–rubber composites and the alignment of the CFs, their trends with the variation of magnetic flux density are shown in Figure 8. It is observed that the thermal anisotropy factor (the thermal conductivity in through-plane direction is divided by that in in-plane direction) has a positive correlation with the alignment degrees of the CFs. With the change in magnetic flux density, they display almost the same trend, which further indicates the relations among the magnetic field, alignment, and thermal properties.

**Figure 9:**
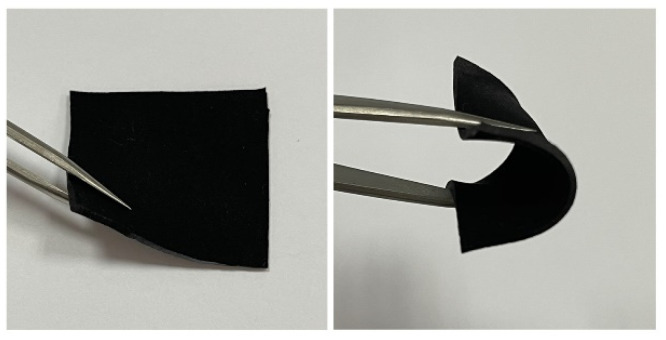
Optical photographs of the CF–rubber composites.

**Figure 10:**
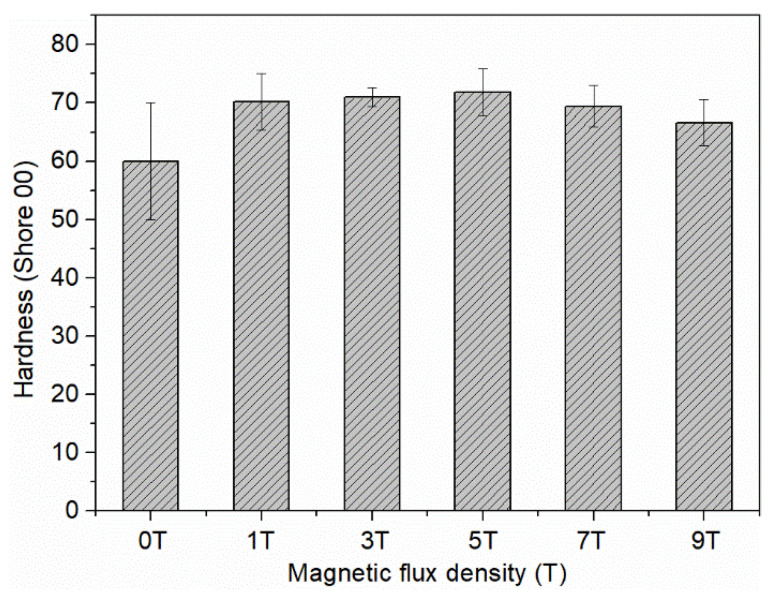
The hardness (shore 00) of the CF–rubber composites applied with different magnetic flux density.

Hardness is an important mechanical property of TIMs. As shown visually in Figure 9, the CF–rubber composites are pliable and very easy to bend and deform. The hardness of the composites is displayed in Figure 10, and is only 60~70 (shore 00). The low hardness benefits from the low filler loading (only 20 vol%) and the discontinuous structure (milled CFs). Furthermore, as a result of the aligned CFs, the composites are capable of efficient heat conduction. Thus, it has great potential as TIMs to fill the gaps between the contact surfaces and enhance the heat transfer at the interface simultaneously. The magnetic flux density has a slight effect on the hardness of the CF–rubber composites, which can be attributed to the magnetic alignment and the anisotropic mechanical property of CFs. This is consistent with the previous study on the Young’s modulus of anisotropic polymer composites [39].

### 3.3. Thermal Management Performance of the CF–Rubber Composites

**Figure 11:**
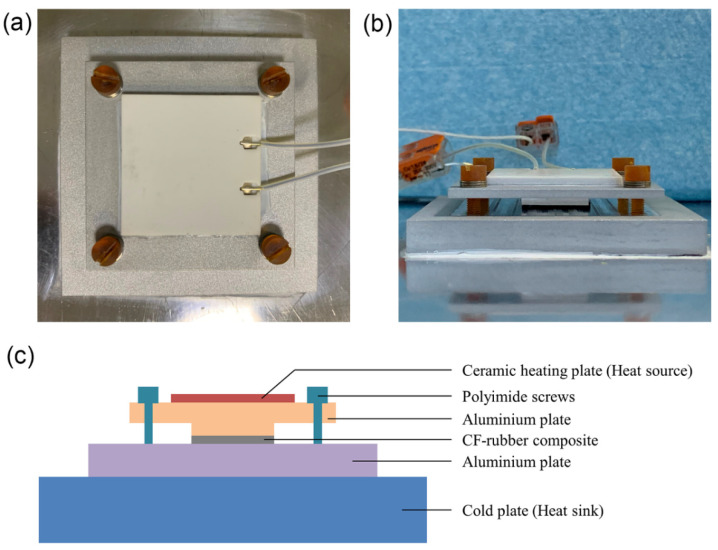
Optical photographs and schematic diagram of the test system. (**a**) Top view; (**b**) front view; (**c**) schematic diagram.

**Figure 12:**
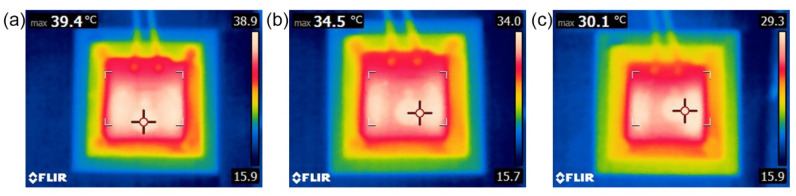
Infrared thermal images of the test system with different CF–rubber composites. (**a**) Sample—0 T; (**b**) Sample—5 T; (**c**) Sample—9 T.

To further evaluate the thermal management performance of the CF–rubber composites in practice, they were used as TIM to transfer heat from the heat source to the heat sink. As shown in Figure 11, a ceramic heating plate was stuck to an aluminum plate with a lug boss of 2.25 cm^2^. The power of the heating plate was set as about 7 W, and thus the heat flux through the lug boss was about 3 W/cm^2^. Another aluminum plate was stuck on the cold plate, chilled by circulating cooling water at 15 °C. A CF–rubber composite of the same size as the lug boss was sandwiched between the two aluminum plates. The detailed dimensions of the entire test system are provided in Table S1. To ensure tight fit, the upper plate and lower plate were connected by screws that were made of polyimide instead of steel to guarantee that most heat would be conducted through the composites rather than the screws. The CF–rubber composites exposed to different magnetic flux densities (0 T, 5 T, 9 T) were used as TIM and tested, respectively. After equilibrium was attained, the surface temperatures were recorded by an infrared thermal imager, as shown in Figure 12. It can be seen that the maximum equilibrium temperature of the 9 T sample was only around 30 °C, which is significantly lower than the 5 T sample (about 35 °C) and the 0 T sample (about 40 °C). On the basis of these results, it can be concluded that the composite at higher magnetic flux density in this study has better thermal management performance, which coincides with the alignment degree of CFs and the thermal conductivities of composites. This means that magnetic field treatment helps the CF–rubber TIMs conduct heat at a faster rate, and then heat generating devices, such as electronic components, can work at a lower temperature, which would benefit the life and reliability of devices.

## 4. Conclusions

In summary, the method of magnetic alignment was used in this study to fabricate CF–rubber composites with ordered and discontinuous microstructure that inherited anisotropic properties from the CFs. The relationships among the magnetic field density, microstructure, and properties of CF-reinforced composites were studied, which have rarely been explored in previous studies. It was observed that the magnetic flux density has a significant effect on the alignment of CFs, and then affects the properties of composites. The CF–rubber composites with high magnetic field treatment not only show strong anisotropy with significantly enhanced through-plane thermal conductivity (11.76 W/(m·K), almost 6 times higher than that of the composite without magnetic alignment), they also demonstrate low hardness (60~70 (shore 00)) with low filler loading and a discontinuous microstructure. Therefore, when used as TIMs, the composites exposed to higher magnetic flux density could effectively reduce the maximum equilibrium temperature of heat source, suggesting them as promising candidates for applications in thermal management for high-power electronic components. This work provides a simple and effective solution for simultaneously improving the thermal conductivity and hardness of thermal interface materials.

## Figures and Tables

**Table 1:** The alignment degree of CFs (*δ*) in CF–rubber composites.

**Magnetic Flux Density (T)**	0	1	3	5	7	9
***δ* (%)**	3.34	2.53	11.64	19.10	54.21	97.38

## Data Availability

The data presented in this study are available on request from the corresponding author.

## Supplementary Materials

The following supporting information can be downloaded at: https://www.mdpi.com/article/10.3390/ma15030735/s1, Table S1: The detailed size of the test system.
