# Supplementary material for: High-Performance Thermal Interface Materials with Magnetic Aligned Carbon Fibers
Source: Materials (Basel). 2022 Jan 19;15(3):735. doi: 10.3390/ma15030735 (PMC8836848; doi:10.3390/ma15030735)
Supplement: Supplementary file 1 [file materials-15-00735-s001.zip › materials-1530525-supplementary.pdf]

Supplementary materials

High Performance Thermal Interface Materials with Magnetic Aligned Carbon Fibers

Qi Wu [1], Jianyin Miao [1,*], Wenjun Li [1], Qi Yang [1], Yanpei Huang [1,2], Zhendong Fu [1] and Le Yang [1]

Theoretical calculation of the alignment time of CF in matrix

Kimura model

$$t = -\frac{6\mu_0\eta}{F(D)\chi_a B^2}\ln\left(\frac{\tan\theta}{\tan\theta_0}\right)$$

with

$$F(D) = \frac{3D\left(-2D\sqrt{D^2-1}+\left(1-2D^2\right)\ln\frac{D-\sqrt{D^2-1}}{D+\sqrt{D^2-1}}\right)}{4\left(D^2-1\right)\left(D^2+1\right)\sqrt{D^2-1}}$$

Where D is the aspect ratio, μ_0 is the magnetic permeability of vacuum ($4\pi\times10^{-7}$T·m/A), η is the viscosity of the matrix, χ_a is the volume anisotropic diamagnetic susceptibility, B is the magnetic flux density, θ and θ_0 are the initial angle and final angle between B and the fiber axis respectively, t is the alignment time. In this work, these parameters are input as follows: D = 15, η = 1/10/100Pa·s, χ_a = 4.0×10^{-6}, B = 0/1/3/5/7/9T, θ = 1°, θ_0 = 90°.

The detailed size of the test system

Table S1. The detailed size of the test system

Component	Number	Size (mm)
Ceramic heating plate	1	30×30×2
Upper aluminium plate	1	45×45×2 (baseplate)

		15×15×3 (lug boss)
Lower aluminium plate	1	60×60×7
CF-rubber composite	1	15×15×1
Polyimide screws	4	Φ3
